# Cultivating Emotional Granularity

**DOI:** 10.3389/fpsyg.2021.703658

**Published:** 2021-12-01

**Authors:** Christine D. Wilson-Mendenhall, John D. Dunne

**Affiliations:** ^1^Center for Healthy Minds, University of Wisconsin-Madison, Madison, WI, United States; ^2^Department of Asian Languages and Cultures, University of Wisconsin-Madison, Madison, WI, United States

**Keywords:** emotional granularity, emotional expertise, mindfulness, Buddhist philosophy, contemplative practice, constructionist theory

## Abstract

An emerging focus in affective science is the expertise that underlies healthy emotionality. A growing literature highlights emotional granularity – the ability to make fine-grained distinctions in one’s affective feelings – as an important skill. Cross-sectional evidence indicating the benefits of emotional granularity raises the question of how emotional granularity might be intentionally cultivated through training. To address this question, we present shared theoretical features of centuries-old Buddhist philosophy and modern constructionist theory that motivate the hypothesis that contemplative practices may improve granularity. We then examine the specific mindfulness-style practices originating in Buddhist traditions that are hypothesized to bolster granularity. We conclude with future directions to empirically test whether emotional granularity can be intentionally cultivated.

## Introduction: Granularity As Emotional Expertise

Emotional expertise involves skills for understanding, experiencing, and regulating emotions (Zeidner et al., 2012; Hoemann et al., 2020). Emotional granularity is an aspect of emotional expertise. It refers to the ability to experience emotions in a precise and context-specific manner (Barrett et al., 2001; Lee et al., 2017). Whereas highly granular individuals make fine-grained distinctions in their emotional experiences, those lower in granularity are unable to do so. For example, those higher in granularity can distinguish feeling angry from other negative feelings, such as feeling fearful, exhausted, or lonely. In contrast, those lower in granularity experience feeling bad without further distinction.

Accumulating evidence from cross-sectional studies suggests that emotional granularity is beneficial. More granular experiences of negative emotions, especially, are consistently associated with better coping and mental health (Kashdan et al., 2015; Smidt and Suvak, 2015). This evidence, along with conceptualizing emotional granularity as a skill, raises the question of how adults can cultivate this expertise. Based on shared theoretical insights in modern constructionist theory and centuries-old Buddhist philosophy, we propose that mindfulness-style practices originating in Buddhist traditions may bolster emotional granularity and that this hypothesis can be empirically tested.

## Theoretical Accounts of Granularity

The idea that emotional granularity is a beneficial skill has emerged relatively recently in psychological science (Kashdan et al., 2015), but it is also a feature of traditional Buddhist accounts of the mind, where it is embedded within a framework that considers granularity of mental states (not just emotions) to be both beneficial and trainable (Dalai Lama et al., 2020). After introducing granularity in each tradition, we focus on shared theoretical features that motivate specific contemplative practices for cultivating granularity.

### Emotional Granularity Is Beneficial: Theoretical Context

Whereas the constructionist theory discussed here largely emerged in the context of empirical investigation over the past 150years (Gendron and Barrett, 2009), Buddhist theories emerged more than 2000years ago. To avoid elevating one discourse over the other, we begin by illustrating how insights regarding the benefits of granularity arise in each framework.

#### Psychological Construction Framework: Granularity Underlies Situated Action

Psychological construction approaches to emotion assume that emotions are constructed events rather than fixed, essential entities (Barrett and Russel, 2015). Within this “family” of theories (Barrett and Russel, 2015), the Theory of Constructed Emotion (TCE; Barrett, 2017) addresses the functionality of granularity. Consider feeling afraid when a fire erupts in one’s house and feeling afraid when giving a speech. In the former, swiftly escaping the house is appropriate and necessary to avoid life-threatening danger. However, fleeing is not helpful in the context of public speaking. Different, situated actions are necessary for effective responding (Barrett, 2013; Wilson-Mendenhall et al., 2013; Wilson-Mendenhall, 2017). In this context, interpreting physiological arousal as a sign that one is ready to engage and perform, instead of signaling a threat to avoid, is beneficial (Jamieson et al., 2018). Granular, context-sensitive processing is necessary to engage in the *specific* actions that will be of benefit in the particular situation,^1^ including actions taken to successfully regulate emotions (Aldao, 2013; Bonanno and Burton, 2013).

#### Buddhist Frameworks: Granularity Enables Insight and Enhanced Regulation

Although “emotion” is not a superordinate category used by Buddhist theorists, the capacity for experiential granularity – the careful parsing of one’s mental states – is a highly valued skill cultivated by meditative practices beginning with the early *Abhidharma*^2^ literature. This emphasis on granular accounts of experience emerges from the perspective that ordinary persons are largely unaware of – or mistaken about – many phenomenally accessible aspects of experience, and this lack of insight into one’s own experience perpetuates suffering (Dalai Lama et al., 2020). Enhanced experiential granularity enables insight into experience in ways that relieve suffering, and it also enables one to more carefully regulate aspects of experience, such as attention and affect (Anālayo, 2003, 2018; Dalai Lama et al., 2020).

### Shared Theoretical Features

Table 1 specifies shared theoretical features across the TCE and the Dharmakīrtian Revision of the Buddhist Abhidharma that motivate the hypothesis that contemplative practices improve emotional granularity. These features include (1) top-down construction, (2) granular concepts, and (3) goal-directed outcomes.

**Table 1:** Shared features that motivate why specific contemplative practices may be effective for cultivating emotional granularity.

Shared feature	Psychological Science *Theory of Constructed Emotion*	Buddhist Philosophy *Dharmakīrtian Revision*	Function of Contemplative Practices Grounded in Buddhist Traditions
***Feature 1*** Because emotions are constructed, emotional habits can be disrupted	Emotional experiences are constructed through active inference. Prior experiences are reinstated (i.e., “concepts”) to categorize sensory input such that the brain understands what caused the sensations and how to act. Emotional habits emerge *via* this top-down construction. Habits lacking granularity can result in ineffective action that does not address the situation at hand (e.g., avoidance coping). Due to the constructed nature of these habits, however, they can be transformed.	Categories of mental states appear to exist as real things in the world, but they are actually constructed through the process of concept formation. Prior experience shapes the concepts deployed in a given context, and that concept in turn shapes one’s behavioral response, prompting certain behaviors while inhibiting others. Through training, one can come to recognize that concepts are constructed in this way and learn to revise them, despite prior conditioning.	*Acceptance*, *decentering*, and *dereification* practices disrupt emotional habits. Instead of avoiding feelings (especially distress), *acceptance* and *decentering* encourage observing emotional experience from a nonjudgmental, impartial perspective without deploying habitual conceptualizations. *Dereification* that involves experiencing emotions as dynamic, constructed mental states would, in theory, disrupt sensorimotor inferences and make it possible to construct experience differently.
***Feature 2*** Emotions can be transformed through granular concepts	Because emotions are constructed, emotional experiences can be transformed through concept construction. Precise emotion word labels, as well as language that specifies situational details, are tools for constructing granular concepts that serve a particular goal-based function, with categorization instantiating context-specific action (and regulation) to navigate the situation at hand.	Since categories of mental states are constructed through concept formation, they can be radically revised, with that revision driven especially by the efficacy of the concepts to achieve context-specific goals. Experiencing conceptual contents as mental constructs facilitates this revision, as does careful parsing of the ways that the concepts illuminate or obscure features of a given mental state.	*Decentering* and *noting* practices use labeling to precisely parse one’s mental state as it changes from moment to moment. *Dereification* underlies the realization that one’s emotional experience is one of many possible constructions. Thus, it invites exploring alternate constructions and observing how they unfold (*via acceptance*, *decentering*, *noting*).
***Feature 3*** Goals shape the outcomes of granularity	Granular categorization facilitates goal-relevant outcomes. These outcomes may not be beneficial to oneself and/or others if the goal (i.e., the purpose of categorization) is not aligned with well-being.	Concepts function to enable goal-directed behavior, and a concept’s efficacy depends on its ability to accurately predict success. The goal itself, however, may not be conducive to the elimination of suffering, and goals must also be a focus of analysis.	Just as interventions derived from Buddhist practices promote various models of well-being, Buddhist practices are embedded within the larger context of relieving suffering, which is taken as a normative goal for all Buddhist traditions.

#### Shared Theoretical Features in the Theory of Constructed Emotion

The TCE specifies how the brain constructs emotions (Barrett, 2017). Whereas several models examine granularity in processing that occurs after an emotion emerges [e.g., *via* “identification” (Gross, 2015), “feelings-as-information” (Schwartz, 2011), or “regulation flexibility” (Pruessner et al., 2020)], the TCE characterizes granularity during the dynamic process of constructing an emotional experience.

The TCE is grounded in a predictive (vs. reactive) model of brain function (Barrett and Simmons, 2015; Chanes and Barrett, 2016; Barrett, 2017). In brief, the brain predicts forward in time to prepare for movement and anticipate the body’s energy needs. Prior experiences are reinstated to predict the cause of incoming sensory changes, and the visceromotor changes and motor actions required to deal with that causal occurrence (Hoemann et al., 2019). This top-down prediction is confirmed or corrected by bottom-up sensory input. Once a prediction is confirmed, sensory input is categorized such that the brain understands what caused the sensations and how to act. This active inference constructs emotional experiences (and other mental states). Although implicit emotional habits often stabilize *via* this top-down categorization, we propose that, due to their constructed nature, emotional habits lacking granularity can be transformed (Table 1, feature 1).

“Concepts” is another name for the brain’s predictions (i.e., its internal model; Hoemann et al., 2019). Because emotions are constructed, they can be transformed by altering concepts (Table 1, feature 2). The TCE points to language as a tool for granular concept construction (Barrett et al., 2007; Lindquist et al., 2015; Hoemann et al., 2019). An emotion word like “angry” constructs concepts that integrate body and world to serve a particular goal-based function, such as overcoming an obstacle (Hoemann et al., 2019).^3^ Grounded in a situation, this goal-based function facilitates specific actions (e.g., protesting injustice or confronting a partner). Discrete emotion categorizations (e.g., angry, afraid, or sad) thus serve to navigate negative affect in the situation at hand (Barrett, 2013), which can include context-sensitive regulation (e.g., relationship repair after an angry argument; Barrett et al., 2014). Without such categorizations, indistinct and ineffective action may be repeated across situations involving negative affect, such as avoidance coping. Precise language further refines categorization (e.g., as annoyed, resentful, or furious) to tailor action and regulation in the situation (e.g., “letting go” of a minor annoyance). Moreover, language can specify detail such that actions (including regulation) become increasingly situated. For example, noting one’s fatigue during an angry argument may serve to initiate rest before respectfully reengaging. Such details can also shift categorization. Noticing fatigue initially, for example, may shift categorization such that anger is not experienced.

In the TCE framework, granularity facilitates goal-relevant and culturally congruent outcomes (Hoemann et al., 2020). If the goal – the purpose of categorization – is not aligned with well-being, we propose that granularity may not be beneficial (Table 1, feature 3). During an experience of anger, for example, granular categorization with the goal of regulating one’s intense feelings is more likely to support well-being than granular categorization with the goal of enacting revenge. Goals can also shape the emotion experienced. Instead of experiencing anger toward another, for example, one might experience compassion, if goals shift to recognizing others’ suffering.

#### Shared Theoretical Features in the Dharmakīrtian Revision of the Buddhist Abhidharma

Theories and practices that promote experiential granularity emerge first in the *Abhidharma* literature, possibly dating to Buddhism’s earliest period (5th century BCE). Presenting extensive lists of “mental factors” (Sanskrit, *cetasika*), including elements of attention, affect, and cognition, this literature played – and continues to play – a central role in Buddhist contemplative practice (Anālayo, 2003). These lists provide the initial Buddhist framework for carefully parsing one’s experience, making experiential granularity a target of mental training.

While some *Abhidharma* traditions did not essentialize mental categories (Gethin, 1992; Heim, 2013), others employed an essentialist approach (Westerhoff, 2018) that limits granularity. For essentialists, an experiential feature belongs by virtue of its essence to a particular category (such as “anger”); thus, multiple, context-dependent categorizations of that feature are not possible. In response to this essentialism, the Indian Buddhist philosopher Dharmakīrti (7th century C.E.) promoted an anti-essentialist account of concept formation that enhances granularity. Instead of appealing to essences, Dharmakīrti maintained that concepts are constructed through the triggering of associations with past experience within a goal-oriented framework rooted in the causal capacities of whatever is being conceptualized (Dunne, 2011; Eltschinger et al., 2018; Table 1, feature 1). Thus, when one conceptualizes two experiential features at different times as “anger,” to ordinary persons, it seems that this conceptualization is simply picking out some identical essence in both instances. The two features are actually unique, but a single concept construes them as the same. In short, given the context formed by one’s goals, the process of concept formation ignores those experiences’ individual differences and constructs a concept that picks out their relevant causal features. If reliable, that concept predicts the outcomes of one’s behavior in ways that enable effective action (Dunne, 2004, 2011; Ganeri, 2011). Concepts are thus highly context-sensitive, and they potentially are highly flexible because an experiential feature does not *in essence* belong to any single category; it is instead open to numerous context-relevant conceptualizations (Table 1, feature 2). To attain that flexibility, one must be trained to recognize the process of concept formation and the illusion of essences that it creates, along with recognizing the role played by conditioning from prior experience. Trained in such a way, one can choose to radically revise the categories used to parse experience, such that an instance of “anger” might be re-conceptualized as “hunger,” in the right context. Crucially, since concepts are always formed in relation to goals, the re-conceptualization of experience must occur within a framework of goals that move one along the Buddhist path, whose endpoint necessarily includes the relief of suffering (Dunne, 2015; Anālayo, 2021; Table 1, feature 3).

## Functions of Contemplative Practices Grounded in Buddhist Traditions

One can characterize a contemplative practice as a cultural practice (Hutchins, 2008) that emphasizes self-awareness, self-regulation, and/or self-inquiry for the purpose of self-transformation, with formal, seated meditation serving as a paradigmatic form (Lutz et al., 2007, 2008; Davidson and Dahl, 2017). In Buddhist cultures, mindfulness meditation has for centuries been a prominent contemplative practice that has more recently been adapted to secular interventions (Kabat-Zinn, 2011). As shown in Table 1, several features of mindfulness-style practices, whether in Buddhist or secular contexts, likely train the capacity for experiential granularity, including meta-awareness, decentering, and dereification (Dunne, 2015). One feature is the instruction to remain “non-averse” to experience (Buddhaghosa, 1976). In the style of mindfulness found in Buddhism-derived, secular Mindfulness-Based Interventions (MBIs), this is usually articulated as maintaining an attitude of “acceptance” or “friendliness” toward experience (Bishop et al., 2004; Kabat-Zinn, 2013), along with a “non-reactive” stance (Bernstein et al., 2015). This attitude is crucial because increased granularity requires a close examination of experience, but if one is averse to an experience that one labels as “unpleasant” or “bad,” then one cannot approach and carefully observe that experience to describe it in a more nuanced fashion.

Likewise, the traditional Buddhist emphasis on deliberately parsing experience into categories, which is best exemplified by the “noting” practice promoted by the influential Burmese teacher Sayadaw (2016), also serves to enhance granularity.^4^ The instruction is to “note” whatever occurs in experience through mental verbalization at each moment – such as “planning, planning, planning, pain, pain, pain.” Both traditional and contemporary mindfulness practice include the instruction to not construe mental states as “belonging to me” (Sanskrit, *ātmīya*; Dalai Lama et al., 2020), often articulated in MBIs as “not identifying with” one’s emotions (Bernstein et al., 2015). This “decentering” facet may enhance granularity by providing the psychological distance to deploy descriptions of experience that do not conform with one’s self-concept.

Mindfulness-based interventions often emphasize the need to “let go” of the “story” that one is telling about one’s experience (Kabat-Zinn, 2013), and this reflects more directly a Dharmakīrtian influence. Specifically, Dharmakīrti’s non-essentialist account posits that concepts are mere mental constructs that never fully capture an object’s identity. As such, concepts can be experienced just as mental events, and this contemplative technique – recognizing that thoughts are simply events in consciousness – emerges directly from Dharmakīrtian philosophy in non-dual meditation styles (Brunnhölzl, 2007; Dunne, 2015). In MBIs, this technique is central to dereification (Bernstein et al., 2015; Lutz et al., 2015), and it is crucial for enhancing emotional granularity, since it permits one to set aside habitual conceptualizations that may seem especially “real” or “true” (Dahl et al., 2020). Dharmakīrti’s approach also permits the application of competing concepts to the same experience, and this promotes reappraisal – a technique that became more prevalent in Buddhist practices starting around Dharmakīrti’s time, such as “Mind Training” practices (Jinpa et al., 2006; Dahl et al., 2015; Jinpa, 2015).

## Future Directions

A research agenda emerges from the interdisciplinary integration illustrated in Table 1. Key questions for future research are presented in Table 2. Because only a handful of studies address these questions, we highlight findings from these studies in the context of discussing future directions.

**Table 2:** Questions for future research on cultivating emotional granularity.

**Mindfulness-Based Interventions**	
1.	Do mindfulness-based interventions, such as Mindfulness-Based Stress Reduction, improve emotional granularity?
2.	Which features of MBI practices (if any) contribute to cultivating emotional granularity (e.g., acceptance, decentering, dereification, noting)?
**Hybrid Interventions**	
3.	Do hybrid interventions that include language-based categorization of emotions, such as Mindfulness-Based Cognitive Therapy (MBCT), provide a more comprehensive approach to cultivating emotional granularity?
4.	What novel, hybrid interventions may be effective in cultivating emotional granularity, especially in the context of preventing (vs. treating) psychopathology?
**Emotional Granularity as Mediator**	
5.	Does training-related improvement in emotional granularity mediate beneficial changes in emotion regulation (e.g., decreased use of maladaptive coping strategies)?
6.	Is training-related improvement in emotional granularity a mediator of beneficial changes in mental health (e.g., decreased mood disorder symptoms) and sustaining those changes (e.g., reduced relapse)?
**Methods and Measurement**	
7.	What forms of emotional granularity are overlooked in current measurement approaches?
8.	Does measuring goals help distinguish when emotional granularity is beneficial?

### Mindfulness-Based Interventions

Do secular MBIs improve emotional granularity? To our knowledge, only one MBI study has examined emotional granularity as it is typically measured *via* experience sampling. This study demonstrated that improved granularity of negative emotions following Mindfulness-Based Stress Reduction was mediated by acceptance and decentering skills, even when controlling for changes in negative affect (Van der Gucht et al., 2019). Consistent with Table 1, this finding suggests that emotional granularity may improve through engaging with negative experiences from a more impartial, precise perspective, without experiential avoidance. Because this relatively small study did not include a control group, the results need to be replicated in larger, randomized controlled trials (RCTs).

As Van der Gucht et al. showed and as posited in Table 1, it is important to investigate whether specific features of contemplative practice cultivate emotional granularity (e.g., acceptance, decentering, dereification, and noting). Moreover, fine-grained neuroscientific accounts of how each feature contributes to enhancing emotional granularity would be valuable. To develop such accounts, it may be fruitful to integrate the constructionist model described here with relevant facets of the growing literature on mindfulness and emotion regulation, such as using awareness practice to expand beyond a narrow focus on threat and attend to other situational features (Hill and Updegraff, 2012; Roemer et al., 2015).

### Hybrid Interventions

Mindfulness-based interventions are increasingly being integrated with other intervention approaches (Hayes et al., 2011; Renna et al., 2017). Evidence suggests, for example, that Mindfulness-Based Cognitive Therapy reduces risk of depressive relapse for those with recurrent depression (Kuyken et al., 2016; Segal et al., 2018). Based on Table 1, integration of the language-based categorization of emotional experiences involved in cognitive therapy (Beck and Haigh, 2014) with MBI practices would be a strong approach to cultivating the emotional granularity that may sustain mental health. Is the coupling of these approaches more impactful in bolstering beneficial emotional granularity than either alone?

Nonclinical populations may also benefit from integrating language-based approaches that expand the range and context-sensitive use of emotion vocabulary (Kashdan et al., 2015). We propose that situated learning is necessary to construct concepts that navigate the situation at hand (Lebois et al., 2020). Consistently labeling an emotion in a particular situation is thought to establish coherent concepts that implement context-specific, goal-directed actions (Hoemann et al., 2019). It is an open question whether integrating MBI practices with vocabulary expansion would be particularly impactful for cultivating emotional granularity.

### Emotional Granularity as Mediator

It will be important to ascertain whether training-related increases in emotional granularity are beneficial and thus to consider emotional granularity in relation to other mechanisms of change. Cross-sectional studies suggest that experiencing negative emotions with greater granularity is associated with less maladaptive coping, such as binge drinking, aggression, and self-injurious behavior (Kashdan et al., 2015). These findings suggest that training-related increases in emotional granularity may mediate improvements in emotion regulation. Higher granularity of negative emotions is also related to fewer symptoms of depression and anxiety (Demiralp et al., 2012; Kashdan et al., 2015), which prompts the question of whether training-related increases in emotional granularity may mediate improved and sustained mental health.

### Methods and Measurement

Dismantling RCTs experimentally manipulate elements of an intervention to systematically investigate their impact. Consistent with Van der Gught and colleagues’ finding that acceptance mediated changes in emotional granularity, recent dismantling RCTs suggest that acceptance is an “active ingredient” underlying the affective benefits of MBIs (Lindsay and Creswell, 2019). This approach is promising for examining how features of contemplative practice may shape emotional granularity (e.g., acceptance and dereification), as well as how other approaches (e.g., cognitive therapy and vocabulary expansion) may interact with contemplative practice to cultivate emotional granularity.

Measures of emotional granularity primarily focus on differentiation of same-valence categories, such as fear, sadness, and anger (Kashdan et al., 2015; Smidt and Suvak, 2015). Buddhist traditions draw attention to the partitioning of emotion, cognition, and perception in Psychology. Dissolving such superordinate categories suggests measuring other forms of fine-grained granularity, including precision within the aforementioned emotion categories (Erbas et al., 2019), sensitivity to dimensions of emotional “thought” such as ruminative repetition (Nolen-Hoeksema et al., 2008; Watkins, 2008), and nuance in relation to bodily “perception” (e.g., identifying hunger as contributing to anger). Moreover, Table 1 suggests that measuring goals may be important for distinguishing beneficial granularity. Developing precise approaches for capturing granularity and the situated actions enabled by that granularity is an important future direction.

## Concluding Remarks

In conclusion, we posit that “deep integration” between constructionist approaches in affective science and scholarship in Buddhist traditions can stimulate novel research (Wilson-Mendenhall et al., 2019). Theory and initial research bolster the hypothesis that contemplative practices contribute to cultivating beneficial emotional granularity, a claim that can be empirically tested.

## Data Availability Statement

The original contributions presented in the study are included in the article/supplementary material, and further inquiries can be directed to the corresponding author.

## Author Contributions

CW-M and JD outlined the manuscript together, each wrote specific sections as designated during the outlining process, and contributed to revising the manuscript. CW-M led a major revision to the manuscript based on helpful reviewer comments. All authors contributed to the article and approved the submitted version.

## Conflict of Interest

CW-M has served as a consultant to the nonprofit organization Healthy Minds Innovations.

The remaining author declares that the research was conducted in the absence of any commercial or financial relationships that could be construed as a potential conflict of interest.

## Publisher’s Note

All claims expressed in this article are solely those of the authors and do not necessarily represent those of their affiliated organizations, or those of the publisher, the editors and the reviewers. Any product that may be evaluated in this article, or claim that may be made by its manufacturer, is not guaranteed or endorsed by the publisher.

## Footnotes

^1^Even the act of fleeing physical danger (“flight”) benefits from situational granularity. In fire safety training, for example, one practices getting low and going under smoke to an exit as “flight.”

^2^In Pali, the canonical language of the Theravāda tradition, the equivalent term is *Abhidhamma*.

^3^Events categorized as anger or any other discrete emotion (e.g., fear, sadness, and joy) vary widely in their features, including the context-dependent actions that facilitate goal-relevant outcomes. These situational instances can be constructed without any categorical “essence.”

^4^Despite the traditional context of Burmese Vipassanā meditation and lacking direct contact with the DharmakÄ«rtian approach, Mahāsi Sayadaw nevertheless promoted a “noting” practice that is not constrained by such lists (Sayadaw, 2016). To the extent that MBIs encourage careful observation of experience, they also do so without normative categories.
